# Violence against men in intimate partnerships in Dédougou, Burkina Faso: reported experiences, forms, and the culture of silence

**DOI:** 10.3389/fsoc.2026.1888378

**Published:** 2026-10-16

**Authors:** Yacouba Tengueri

**Affiliations:** Department of Sociology and Anthropology, Université Daniel Ouezzin Coulibaly, Dédougou, Burkina Faso

**Keywords:** Burkina Faso, domestic violence, intimate partner violence, male victimisation, masculinity

## Abstract

**Background:**

Domestic violence research has largely focused on women as victims, leaving male victimisation under-recognised. This study examines the prevalence, forms, and consequences of domestic violence against men in Dédougou, Burkina Faso.

**Methods:**

A mixed-methods design combined a KoboToolbox survey of 598 men with a current or past intimate relationship with 26 semi-structured interviews with male victims, women, religious and customary leaders, law enforcement, the judiciary, and social services (June–July 2025). The survey used non-probability convenience sampling, so the sample is not statistically representative of men in Dédougou or in Burkina Faso.

**Results:**

64.55% of surveyed participants reported experiencing at least one form of violence, most often verbal (49.50%), psychological (24.58%), economic (21.74%), sexual (11.54%), or physical (9.20%). The principal factors reported by respondents were economic difficulties (59.20%) and jealousy or infidelity (52.51%). Only 0.78% of those reporting violence sought help from law enforcement, while 43.26% stayed silent. Consequences included anxiety (45.65%), low self-esteem (31.27%), and social isolation (25.59%).

**Conclusion:**

Domestic violence against men is common but under-recognised in Burkina Faso. Findings call for inclusive policies, formal recognition, support services, and awareness campaigns, without diminishing the gravity of violence against women.

## 1 Introduction

Domestic violence, defined as the occurrence of violence by one partner upon the other (Halouze et al., 2020), encompasses aggression, threats, and verbal, physical, sexual, and economic coercion (Gruev-Vintila and Toledo Candia, 2021). Such violence is recurrent or liable to recur, and causes harm to the integrity of the other person (Halouze et al., 2020). The intentional character of the act of violence, the objective of which is to inflict pain or physical injury upon another, is fundamental to its characterisation. Furthermore, a distinction must be drawn between domestic violence and marital conflict. Domestic violence differs from marital conflict in that it lacks bilateral freedom of expression and equality of standing (Vasseur et al., 2020). Historically, the concept of ‘domestic violence’ was systematically associated with ‘battered women’ (Dobash and Dobash, 1979; Walby and Allen, 2004), and was itself reduced to physical violence alone (Nasr, 2009). Since then, the term has come to encompass other forms of violence and has progressively acknowledged the victimisation of men (Bates, 2019, 2020; Hine et al., 2020, 2022).

Research on IPV has historically centred on women as victims and men as perpetrators, reflecting both the scale of violence against women and the priorities of early advocacy and scholarship. More recent work increasingly recognises a wider range of victim–perpetrator dynamics, including bidirectional violence and male victimisation within intimate partnerships. Acknowledging these under-recognised forms of IPV does not minimise violence against women; on the contrary, it makes definitions, measurement, and response strategies more complete and inclusive. Gueta and Ben-Porat (2023), for example, note that early IPV research, built around notions of ‘wife abuse’ and ‘violence against women’, largely overlooked the victimisation of men and boys, and call for conceptual models that take masculinities and the gender-specific barriers men face when seeking help into account. Rollè et al. (2019) similarly argue that research needs to move beyond a rigid perpetrator–victim binary to capture the full diversity of violent dynamics, pointing to studies of IPV in same-sex couples (Rollè et al., 2018) and among older partners (Gerino et al., 2018) that show IPV cuts across gender, sexual orientation, and age.

The invisibilisation of domestic violence against men is linked to the patriarchal system that legitimises male dominance (Bourdieu, 1998). The recent works of Dallaire (2001), Hines and Douglas (2010), and Dutton and White (2013) shed light on the existence of violence against men within intimate partnerships; even so, the phenomenon remains minimised and overlooked in scientific research and in the media. Three elements justify the present study. First, violence experienced by men remains substantially under-documented within reporting and support systems in West Africa, which have historically been designed with female victims in mind (Dallaire, 2001; Gaget, 2015; Salomé and Salomé, 2020). Second, masculinity norms that valorise strength and self-sufficiency may limit men’s disclosure of victimisation and their willingness to seek help, a pattern noted across diverse settings (Romito, 2006; Sauvage, 2017). Third, empirical data specific to Burkina Faso on this question remain lacking. As Dallaire (2002) observes, domestic violence is, for those who experience it, a lived reality that can arise from a cycle of mutual escalation between partners, which underlines the value of documenting men’s experiences without adjudicating a broader debate about the relative extent of violence against women and against men.

Within sub-Saharan Africa, the predominance of the patriarchal system and the high masculinity index mean that expectations regarding male masculinity are considerable and vary according to cultural context (Ndiaye, 2021). Indeed, men are presented as the custodians of strength and violence, tasked with protecting the family. In these patriarchal societies, a man must be strong, powerful, dominant, and restrained, whilst women, or the so-called weaker sex, are reduced to a fragility requiring protection. Society expects submission and obedience from women. This social and cultural construction of roles and expectations renders the reversal of violence a social pathology. A woman guilty of violence is perceived as deviant; she is accordingly excluded, stigmatised, and disqualified (Goffman, 1963) from the matrimonial marketplace. By contrast, male violence against women is perceived as normal and tolerated (Nasr, 2009). The societal expectations placed upon men mean that any challenge to this established social order entails serious somatic, psychological, and social consequences for men (Abed et al., 2016) and remains a taboo subject (Collectif anti-masculiniste Île-de-France, 2016). Consequently, being dispossessed of, or lacking, the attributes of masculinity serves to devalue the status of manhood (Kopp et al., 2008; Nasr, 2009). Male victims of domestic violence retreat into silence, worn down by a second form of violence in the shape of society’s denial and intolerance.

This article on domestic violence against men in the municipality of Dédougou does not seek to challenge the documented inequality in violent dynamics between the sexes. Its principal objective is to document the forms, causes, and socio-cultural consequences of domestic violence against men within a patriarchal and masculinist society characterised by denial and non-recognition of the phenomenon. Indeed, according to Abed et al. (2016), societal concern; since men are frequently regarded as perpetrators, male victims of domestic violence remain poorly studied.

Burkina Faso, and the municipality of Dédougou in particular, offer an important setting for such research, given its strong patriarchal norms and expectations of masculinity, pervasive stigma, and a deeply rooted culture of silence, all of which create substantial barriers to disclosure and help-seeking, while formal IPV services remain scarce and oriented almost exclusively toward female victims. By documenting male victimisation in this resource-constrained West African setting, the study speaks to broader IPV strategies around the world, including surveillance, reporting pathways, psychosocial support, legal response, and the design of culturally appropriate, gender-inclusive services. Similar concerns emerge from recent work on low- and middle-income countries, where Landa-Blanco and Mejía Sánchez (2025) note that male victims remain a largely invisible demographic, citing meta-analytic evidence covering 58,357 men (2010–2022) in which roughly one man in five reported physical partner violence, over four in 10 reported psychological violence, and 7% reported sexual violence, figures that point to both the scale and the neglect of the phenomenon in contexts comparable to Burkina Faso.

## 2 Methodology

### 2.1 Study area

This research was conducted in the urban municipality of Dédougou, situated in the Boucle du Mouhoun region in the north-west of Burkina Faso. According to the General Population and Housing Census (Institut National de la Statistique et de la Démographie, 2019), its resident population is estimated at 123,934 inhabitants, comprising 60,555 men and 63,379 women. The municipality of Dédougou is organised into seven (7) sectors and 35 villages. The region is home to several ethnic groups, including the Bwaba, San, Dafing, Fulani, Nouni, and Gurunsi peoples, amongst others. The population and household figures cited in this article refer to the municipality as a whole and are given for context only; data collection was confined to the seven urban sectors, and the 35 villages were not covered.

### 2.2 Study population

The study population comprised men and women currently or previously in an intimate relationship residing in the urban area of Dédougou (its seven sectors). Key informants included representatives of the gendarmerie, the police, the judiciary, the social services (provincial directorate for gender and social welfare), and customary and religious authorities.

### 2.3 Data collection tools and techniques

Two types of data collection instrument were developed for the purposes of this study: a questionnaire and two interview guides. The questionnaire was designed on the KoboToolbox platform and was administered exclusively to men. The questionnaire covered socio-demographic characteristics and the experience of five forms of violence, each measured through behaviourally specific questions covering verbal violence (insults, humiliating or degrading remarks, public denigration), psychological violence (threats, intimidation, controlling behaviour, emotional blackmail), economic violence (confiscation of income, denial of access to household resources, sabotage of economic activities), sexual violence (sexual intercourse or acts imposed without consent), and physical violence (slaps, blows, bites, scratches, and other forms of assault). For each form, respondents indicated whether they had experienced it within their current or most recent intimate relationship, along with its frequency and duration, so the reference period was the duration of the relationship rather than a fixed recall window. The items were developed by the author on the basis of the literature on intimate partner violence, as no pre-existing validated scale was available for this context; the questionnaire was therefore not adapted from a standardised instrument. It was reviewed with a small number of respondents prior to full deployment to check clarity and cultural appropriateness, and it was administered in French, with oral translation into Dioula or Bwamu offered to respondents who preferred a local language. A respondent was classified as a victim if he reported at least one occurrence of any of the five forms of violence within his current or most recent intimate relationship; the sample of 386 victims accordingly includes single, divorced, and widowed respondents who reported such experiences in a relationship that has since ended, alongside respondents currently in a relationship. The distinction drawn in the Introduction between domestic violence and marital conflict (Vasseur et al., 2020) was likewise not separately operationalised in the questionnaire items. Respondents were asked whether specific violent behaviours had occurred, and with what frequency and duration, but no item measured the asymmetry of power between partners, the presence of coercive control, the intentionality of the act, or whether the behaviours were unidirectional or reciprocal. The interview guides were administered to men, women, the gendarmerie, the police, the judiciary, customary authorities, and religious leaders. This mixed-methods approach was intended to capture both the extent of the phenomenon and the complexity of domestic violence against men as an invisible and sensitive subject. Individual semi-structured interviews were conducted with these various actors. This type of interview enables the researcher to apprehend the perceptions, interpretations, and experiences of respondents whilst maintaining sufficient focus to avoid straying from the research objectives (N’Da, 2006).

### 2.4 Sampling and sample

Qualitative sampling was conducted on the basis of purposive selection. The inclusion criteria were based upon the capacity of respondents to provide in-depth information on the issue of domestic violence. These criteria led to the identification of key actors, including the gendarmerie, the police, the judiciary, the social services, and customary and religious authorities. As actors at the heart of the administrative and social management of domestic violence cases, they are well placed to provide an informed and contextualised perspective on the phenomenon, given its invisibility and complexity. In addition to these key informants, male victims of violence identified in the course of quantitative data collection were purposively invited to take part in an individual interview so as to document first-hand accounts of the phenomenon; four such interviews were conducted (Respondents 23 to 26 in the account below). The 11 women interviewed were purposively selected within the community, independently of the male participants surveyed, to provide a broader perspective on perceptions of domestic violence against men and on the social reception of violence attributed to women; they were not necessarily partners of the men surveyed, and this is made explicit wherever a woman’s account is reported.

With regard to quantitative sampling, the target population was all men of marriageable age living in Dédougou who were, or had previously been, in an intimate relationship. The study employed a non-probabilistic convenience sampling design: participants were recruited on the basis of availability and accidental encounters rather than random selection. Within this non-probabilistic framework, geographical balance was pursued by allocating enumerators to each of the seven (7) sectors of the municipality in rough proportion to their population, so that men were approached, and interviewed on the spot if willing and available, across all sectors rather than in a single locality; this improves the geographical spread of the sample but does not make the underlying selection of individual respondents random or probabilistic. The aim was to reach as many households as possible from amongst the 25,742 households recorded in the (Institut National de la Statistique et de la Démographie, 2019). Whilst this convenience-sampling technique does not guarantee perfect statistical representativeness, it remains appropriate in contexts where constraints of time, human resources, or the availability of individuals limit the application of more rigorous methods (Sampieri, 2014). Because a non-probability convenience sample was used, these results cannot be considered statistically representative of all men in Dédougou, still less of all men in Burkina Faso. Men who were more available, more accessible to enumerators, or more willing to discuss their intimate relationships may be over-represented, which introduces a possible selection bias. The prevalence figures reported below should therefore be read as descriptive of the surveyed sample rather than as estimates for the wider population.

### 2.5 Data processing and ethical considerations

Two types of analysis were employed in this study. For qualitative data, thematic content analysis was adopted. Interviews were audio-recorded where participants consented, or captured through detailed field notes, and were subsequently transcribed and analysed using Microsoft Word 2019. Coding was performed by the author using a primarily inductive approach, with themes (including the culture of silence, forms of violence, and help-seeking) developed iteratively from the codes rather than derived from a pre-existing coding frame; as coding was carried out by a single analyst, no inter-rater reliability statistic is available, which is acknowledged as a limitation in the Limitations section, under ‘Single-coder qualitative analysis’. The analysis addressed both the manifest and latent content of respondents’ discourse. Quantitative data collected via the KoboToolbox platform were exported in XLS format. Prior to analysis, the data were processed before generating statistical tables. A descriptive analysis of domestic violence data was conducted to illustrate the extent of the phenomenon. In order to preserve the anonymity of respondents, each participant was assigned an alphanumeric code. In the absence of a constituted research ethics committee at the author’s institution, the research protocol, comprising the questionnaire and the two interview guides, was submitted for review and approval to the Vice-Presidency in charge of Research of Université Daniel Ouezzin Coulibaly, which acted as the competent institutional authority. The study therefore received institutional approval rather than approval by a formally constituted ethics committee; it is not the case that ethical approval was deemed unnecessary. Consent was obtained orally rather than in writing, and no personal identifier was recorded, each participant being designated solely by an alphanumeric code. This account is restated in full in the Ethics statement below. Access to and conduct of the survey were conditional upon the verbal consent of participants. Participants took part freely and voluntarily. Furthermore, in order to mitigate psychological risks, the administration of both the questionnaire and the interview guides was carried out under conditions of safety and discretion.

## 3 Results

The results of this study are organised around the demographic characteristics of respondents and the forms, frequency, help-seeking behaviour, and socio-psychological consequences of domestic violence against men (Table 1).

**Table 1:** Sample size.

Sampling type	Actor surveyed	Number
Quantitative	Men	598
Qualitative		26
	Imams	2
	Pastors	2
	Customary authority	1
	Gendarmerie	2
	Police	2
	Social Services	1
	Judiciary	1
	Women	11
	Male victims	4
Total participations^1^*		624

Source: Survey, June–July 2025. ^1^*The four male victims interviewed were also among the 598 questionnaire respondents; the total of 624 therefore counts participations and corresponds to 620 distinct individuals.

### 3.1 Socio-demographic characteristics of respondents

Table 2 indicates that 33.61% of respondents were aged between 35 and 44 years, followed by those aged between 25 and 34 years, representing 33.44% of the sample. Respondents aged between 45 and 54 years accounted for 18.56%, whilst those aged 55 years and over and those under 25 years represented 7.36 and 7.02%, respectively.

**Table 2:** Characteristics of respondents.

Variable	Value	Frequency	Percentage (%)
Age	35–44 years	201	33.61
	25–34 years	200	33.44
	45–54 years	111	18.56
	55 years and over	44	7.36
	Under 25 years	42	7.02
Marital status education level	Married (monogamous)	306	51.17
	Married (polygamous)	100	16.72
	Single	72	12.04
	Cohabiting	71	11.87
	Divorced	35	5.85
	Widowed	14	2.34
	Secondary	160	26.76
	Higher	148	24.75
	Primary	122	20.40
	Literate (local language)	93	15.55
	None	75	12.54
Occupation	Trader	140	23.42
	Civil servant	135	22.58
	Farmer	116	19.40
	Artisan	63	10.54
	Tailor	47	7.86
	Mechanic	45	7.53
	Other	52	8.73
Religion	Islam	301	50.33
	Christianity (Catholic, Protestant, etc.)	229	38.29
	Traditional religion	68	11.30

Source: Survey, June–July 2025. Percentages within each variable (Age, Marital Status, Education Level, Occupation, Religion) are calculated on *n* = 598 and may not sum to exactly 100% due to rounding to two decimal places.

The sample comprised 51.17% monogamous married individuals and 16.72% polygamously married individuals. Single respondents accounted for 12.04% of the sample, whilst 11.87% were cohabiting. Divorced respondents constituted 5.85% and widowers 2.34%. The educational attainment of respondents was variable. Indeed, 12.54% had no formal education, compared to 15.55% who were literate in a local language (Dioula, Bwamu). Those with primary and secondary education represented 20.40 and 26.76%, respectively, whilst those with higher education constituted 24.75% of the sample.

Respondents were engaged in a wide diversity of occupations, distributed across approximately 20 categories. The most represented categories in the sample were traders (23.42%), civil servants (22.58%), farmers (19.40%), artisans (10.54%), tailors (7.86%), and mechanics (7.53%). Other occupations, including students, pupils, hairdressers, bricklayers, bus drivers, and security guards, accounted for 8.73% of the sample. Respondents were distributed across three major religious groups. Muslims were the most numerous, followed by Christians (Catholic and Protestant). Indeed, more than half (50.33%) declared themselves Muslim. Christians constituted 38.29% of the sample, whilst 11.30% declared themselves practitioners of traditional religion.

### 3.2 Forms and frequency of domestic violence

This survey revealed that 64.55% of the men who participated in this study had experienced at least one form of violence within their intimate partnership, whilst 35.45% reported having experienced no domestic violence. Among the men subjected to violence, the forms reported were verbal violence (49.50%), psychological violence (24.58%), economic violence (21.74%), sexual violence (11.54%), and physical violence or assault and battery (9.20%). During individual interviews, physical and sexual violence were topics that respondents found particularly difficult to address. The few cases mentioned revealed a profound suffering endured in silence. These exchanges on the subject were marked by emotional charge (lowering of the voice, downcast gaze, silence, suppressed tears). The interviews represented, for some, moments of liberation and relief; for others, they constituted the exhumation of painful memories. Respondent 23 (male victim, in his thirties) described the violence he experienced in the following terms:

During an argument with my wife, she overpowered me in the courtyard. She struck me and bit my genitals. After that, she left the household. I went to beg her to come back because I love her. Today, I have erectile difficulties. She cannot complain, since she is the cause of this condition.

Furthermore, it is evident that men are disproportionately affected by verbal and psychological violence, a finding corroborated by the qualitative data. Indeed, respondents stated:

Table 3 reveals that men are disproportionately subjected to verbal and psychological violence. Indeed, during individual interviews, certain respondents affirmed that ‘a woman’s quiver is her mouth’,^1^ or that ‘if one were to take seriously everything a woman says, one might be driven to kill her’. These proverbs suggest that women do not possess the physical strength to rival men but wield a destructive weapon in the form of language. Such sayings implicitly affirm women’s physical incapacity to perpetrate physical violence against men, whilst positioning speech as a means of self-defence against male aggression. Nevertheless, statistical data reveal that women are capable of inflicting blows and injuries upon men; indeed, 9.20% of the men surveyed acknowledged having been subjected to this form of violence.

**Table 3:** Forms of domestic violence.

Variable	Value	Frequency	Percentage (%)
Situation of violence	Yes	386	64.55
	No	212	35.45
Type of violence	Verbal violence	296	49.50
	Psychological violence	147	24.58
	Economic violence	130	21.74
	Sexual violence	69	11.54
	Physical violence (assault and battery)	55	9.20

Source: Survey, June–July 2025. Percentages for the forms of violence are calculated on the full sample of 598 respondents, not on the 386 respondents classified as victims; a respondent could report more than one form of violence.

Furthermore, the frequency with which such violence occurs was found to vary from one couple to another. It emerges that 32.64% of respondents experienced domestic violence ‘occasionally’, 17.88% ‘frequently’, 17.10% ‘very frequently’, and 32.38% ‘rarely’.

Table 4 indicates that 29.27% of respondents had been experiencing domestic violence for less than 6 months, 31.61% for between 6 months and 1 year, 16.84% for between one and 3 years, and 22.28% for more than 3 years.

**Table 4:** Frequency and duration of domestic violence.

Variable	Value	Frequency	Percentage (%)
Frequency of violence	Occasionally	126	32.64
	Rarely	125	32.38
	Frequently	69	17.88
	Very frequently	66	17.10
Duration of violence	6 months to 1 year	122	31.61
	Less than 6 months	113	29.27
	More than 3 years	86	22.28
	1 to 3 years	65	16.84
Total		386	100

Source: Survey, June–July 2025.

### 3.3 Perceived contributing factors to violence against men

This study highlights a multitude of explanatory factors underlying domestic violence against men. These factors are complex and complementary in understanding the behaviour of both the victim and the perpetrator. For a large majority, 59.20% of respondents, the inability of a man to meet the economic needs of the family may be a primary cause of violence against men within intimate partnerships. Other contributing factors included infidelity or jealousy (52.51%), conflicts involving power or respect within the couple (36.12%), cultural misunderstandings (21.37%), the influence of the wife’s family (21.24%), alcohol and drug consumption (17.22%), witchcraft (8.86%), curses (8.36%), and, at 2.19%, lack of communication, the irascible character of the partner, sexual dissatisfaction, the wife’s negative social influences, forced marriage without love, and violence toward children.

### 3.4 Help-seeking and management of domestic violence

Table 5 reveals that 56.74% (219 of 386) of men subjected to violence by their partner sought the intervention of external actors in managing the crisis. The individuals and bodies involved in seeking solutions to situations of violence included friends or relatives (34.20%), religious authorities (14.77%), the man’s parents (2.33%), the wife’s family (1.30%), witnesses at the marriage ceremony (1.04%), neighbors (0.78%), law enforcement (0.78%), health centers or social welfare structures (0.52%), children (0.26%), matrimonial coaches on social media (0.26%), and an actor not further specified by the respondent (0.52%); this breakdown sums exactly to the 219 respondents (56.74%) recorded as having sought help. Statistical data indicate that men experiencing domestic violence rarely turn to law enforcement or health and social services. By contrast, 43.26% (167 of 386) of men subjected to violence preferred to maintain a posture of silence, their fear of having their situation of subjugation discovered causing them to withdraw into reticence.

**Table 5:** Actors consulted and help-seeking.

Variable	Value	Frequency	Percentage (%)
Help-seeking	Sought help (any actor)	219	56.74
Actors consulted	Friends or relatives	132	34.20
	Religious or traditional authorities	57	14.77
	Parents	9	2.33
	Wife’s family	5	1.30
	Witness at marriage ceremony	4	1.04
	Neighbours	3	0.78
	Law enforcement	3	0.78
	Health centre or social service	2	0.52
	Matrimonial coach (social media)	1	0.26
	Children	1	0.26
	Other / actor not specified	2	0.52
	No help-seeking	167	43.26
Total		386	100

Source: Survey, June–July 2025.

The management of cases involving male victims of violence has consistently been a major concern for victims, their families, state structures, and religious and customary leaders alike. When consulted on cases of domestic violence, religious leaders draw upon Quranic or Biblical passages, or upon the prohibitions enshrined in local customs and traditions, as a means of dissuading women from violating their husbands. Such acts of violence by women are understood to have the social consequence of destroying the future success of their children. For these actors, a woman’s act of violence against her husband is understood to attract a ‘curse’ upon her descendants, since she is expected to be submissive. Indeed, Respondent 10 (Imam, Muslim) stated:

When a man beats his wife, he is asked to apologise and seek forgiveness. But when it is the woman who dominates the man and strikes him, she is made to understand that among us Muslims, this brings a curse upon the success of her children. She must submit to her husband for the sake of her children’s future, as they risk becoming social cases.

This view was shared by numerous female respondents regarding the social consequences of female violence upon children. Society condemns and disqualifies the violent woman. She loses the social attributes of a good wife and mother. Those who free themselves from this social constraint of submission are labelled malicious, witches, or poorly raised, amongst other epithets. Indeed, Respondent 14 (female community member, 52 years old, housewife, Catholic) added:

Violent women are considered wicked, poorly raised, witches. Under normal circumstances, a woman should not be violent, for the sake of her children and their success. A woman should be like a rubbish bin. Today, she refuses to submit, claiming she is emancipated. A woman should be reserved. When a woman is violent, the children are ashamed to introduce her as their mother.

Respondent 13 (Pastor, Protestant) acknowledged the existence of such violence, as he is frequently solicited by his congregation. Indeed, he reported receiving approximately three to four male victims of violence per week. The resolution of conflict is achieved through prayer and counselling for the couple, as he described:

We spend time with them in prayer and provide advice, telling them they must not seek to harm their lives, as every situation has an end. We also help them to understand that conflicts are part of life and that they can be navigated one way or another.

When it proves impossible to reconcile positions within the couple, divorce becomes the ultimate solution for preserving the physical, moral, and mental health of the victimised man. Both families are nonetheless involved in seeking a resolution. In Islam, prior to proceeding to divorce, the partners are given 40 days to find a solution. During this period, they must fulfil all conjugal duties except engaging in sexual relations:

In managing cases of men who are victims of violence, we refer the matter to both families and to customary leaders so that they may offer guidance. If the problem persists and the man requests a divorce, we initiate the divorce process in accordance with religious texts. This process involves granting both the man and the woman 40 days to continue living together whilst observing conjugal duties, except for sexual intercourse. This is to ensure that the woman is not pregnant. However, they must continue to sleep in the same bed as before, without engaging in sexual relations. Even if she undresses, the man must refrain from touching her if he wishes to proceed with the divorce. He must continue to provide the daily food allowance. She is obliged to continue providing his bathing water and preparing his meals throughout the 40 days. If the couple reaches the end of the 40 days without having engaged in sexual intercourse, the divorce is thereby consummated and the woman may return to her family.

Respondent 25 (male victim, divorced) stated:

I divorced 3 months ago; I could not go on, I was at breaking point. No one believed me, because my wife was highly skilled at manipulation, she knew exactly what she was doing. Eventually, it went beyond violence. I chose to file for divorce; she resisted for a year, but it changed nothing. In the end, we signed. My two children are with my mother, and I am currently trying to rebuild myself.

In contrast to religious leaders, state structures proceed by ensuring that the male victim of assault and battery receives medical care at hospital, at the expense of the aggressor, before initiating dialogue to reconcile the parties. Referral to formal proceedings is not automatic when the matter is brought to social welfare services. However, in the event of failure, the case is referred to the police or the judiciary. This is illustrated by Respondent 20 (officer at the regional directorate of social welfare, Dédougou):

When we are consulted, the couple is summoned. Both parties are seen individually to assess the situation and determine whether it is appropriate to refer the case to the police or the judiciary. Case management differs according to the severity of the situation. When there has been assault and battery, the priority is to treat the victim and take them to hospital. We cover the cost of prescriptions. However, if the victim is able to pay for their medication, they are invited to do so. All such expenditure must be reimbursed by the aggressor. It should be noted that the relevant legislation stipulates that cases of assault and battery must be referred to the judiciary; however, in most cases, the parties refuse to go to the police or the courts, and we therefore attempt to find solutions.

This reluctance to resort to the gendarmerie, police, and judiciary is linked to the perception of these institutions as punitive bodies. Recourse to such structures is equated with renouncing reconciliation, which suggests that couples are more oriented toward discreet, conciliatory resolution than toward the formal sanctioning of the other party. Indeed, qualitative interviews shed light on this low recourse to state structures. Respondent 22 (officer at the central police station) stated:

At present, no formal complaint has been filed. People turn instead to social services and the gender directorate. Victims often do not want the matter to leave the police station. The majority of cases we receive concern requests for assistance with reconciliation and improving collaboration. To date, there has been no clear-cut case of a man formally identified as a victim of domestic violence.

For 63.04% of respondents, society does not recognise violence against men, in contrast to 19.40% who believed it does, and 17.56% who had no opinion on the matter. Nevertheless, respondents estimated that men experiencing domestic violence retreat into silence for several reasons, including fear of stigmatisation (64.05%), social pressure to appear strong (52.34%), absence of support structures (30.77%), cultural or religious reasons (24.41%), and, at 5.18%, other reasons such as contempt, ridicule, disbelief, lack of support, and the risk of being labelled a ‘pushover’, a coward, or effeminate. This demeaning gaze toward the victim accounts, in the present context, for what may be described as the ‘culture of silence’ surrounding violence. These figures, detailed in Table 6, are consistent with what the sociological literature terms hegemonic masculinity (Connell and Messerschmidt, 2005): where fear of stigmatisation (64.05%) and social pressure to ‘appear strong’ (52.34%) are the two most frequently cited reasons for silence, admitting victimisation is experienced less as a factual disclosure than as a failure to embody the strength and self-sufficiency expected of men, which is precisely the pressure Respondent 24 (male victim, in his thirties) described directly:

**Table 6:** Explanatory factors for the culture of silence among victims.

Value	Frequency	Percentage (%)
Fear of stigmatization	383	64.05
Social pressure to ‘appear strong’	313	52.34
Absence of support structures	184	30.77
Cultural or religious reasons	146	24.41
Other	31	5.18

Source: Survey, June–July 2025. Percentages are calculated on the full sample of 598 respondents; multiple reasons could be selected, so percentages sum to more than 100%.

If a man speaks out about the violence he is suffering, society will not believe him, and will label him a coward. It is a difficult label to bear, so the man prefers to remain silent and suffer alone, in silence.

The strong internalisation of a dominant, assertive masculinity renders acceptance of violence perpetrated by the so-called weaker sex upon the stronger particularly difficult. A degree of denial regarding the reality of such violence leads men in relationships to withdraw into silence. They endure a dual form of violence, that of their partner and that of a society which struggles to acknowledge their situation.

For some respondents, men are disproportionately subjected to psychological violence because ‘a woman’s strength lies in her tongue’. Were men to lose their composure, they would risk resorting to violence against women, hence the proverb: ‘a woman’s quiver is her mouth’. Yielding to the provocation of a woman’s words leads only to hospital or prison.

This violence is not perceived uniformly by all victims. Certain respondents considered that the violent situation did not reflect the woman’s conscious intention to dominate or demean them, or to invert the power dynamic within the couple. Rather, they perceived the violence as a manifestation of love and consideration. Indeed, Respondent 26 (male victim, Muslim) stated: ‘Men who are beaten are valued; personally, I feel valued by my wife. I know she loves me’.

### 3.5 Social and psychological consequences of domestic violence

Psychological support and mediation are essential in managing situations of domestic violence. Male victims of violence are, for the most part, under considerable stress. Consequently, they take refuge in tobacco, alcohol, or other psychoactive substances as a means of coping with their domestic difficulties. Social disqualification leads some to severe depression and, in the worst cases, to suicidal ideation. For others, abandonment of the household in order to escape their partner becomes the only viable solution. Indeed, Table 7 indicates that male victims of violence are prone to anxiety and stress (45.65%), diminished self-esteem (31.27%), social isolation (25.59%), professional or economic difficulties (12.04%), suicidal ideation (6.69%), and other consequences. These percentages are calculated on the full sample of 598 respondents rather than on the 386 men who reported violence, and respondents could report more than one consequence; they should therefore be read as the share of all respondents reporting each consequence, not as the share of victims (see the note to Table 7).

**Table 7:** Psychological effects of violence and non-disclosure.

Variable	Value	Frequency	Percentage (%)
Effects of violence	Anxiety, stress	273	45.65
	Diminished self-esteem	187	31.27
	Social isolation	153	25.59
	Professional or economic difficulties	72	12.04
	Suicidal ideation	40	6.69
	Other	17	2.84
Reasons for non-disclosure or non-separation	Fear of social judgement	192	27.27
	Shame	178	25.28
	Children	144	20.45
	Love or attachment	131	18.61
	Financial dependence	23	3.27
	Hope of behavioural change	9	1.28
	Desire to save the marriage	8	1.14
	Divorced	17	2.41
	Do not know	2	0.28

Source: Survey, June–July 2025. This table combines two distinct multiple-response items, and the two blocks do not share the same denominator. In the first block (effects of violence), percentages are calculated on the full sample of 598 respondents, from a total of 742 responses; respondents could report more than one effect, and the figures are therefore not proportions of the 386 respondents classified as victims. In the second block (reasons for non-disclosure or non-separation), percentages are calculated on the total number of responses recorded for that item (704 responses) rather than on the number of respondents, which is why they sum to 100%. Each figure in the second block should accordingly be read as the share of reasons given, not as the share of men who gave that reason, and the number of individual respondents behind each category cannot be inferred from the percentage. For the same reason, the percentages in the second block are not comparable with the respondent-based percentages reported in Tables 3, 5, 6, 8 and neither block should be used to derive a prevalence estimate.

These statistical data are corroborated by accounts gathered from law enforcement officers. Respondent 5 (gendarme) illustrated this with the following account:

We were once called in for a case of household abandonment. The man had left everything behind, his wife, his children, and his home, because he could no longer bear the situation. He had rented a room in another neighbourhood, telling his family he was on a mission. He had been absent from the household for over a month. His wife was calling him and he kept saying he was away on assignment. She then contacted a colleague of her husband, who informed her that he was not on any assignment. She put the man under surveillance and tracked him until three o’clock in the morning. He was drinking heavily and moving from bar to bar. She followed him until he parked his vehicle in front of a building and contacted us. When we arrived on the scene, there was nothing, just a mattress. When he came home, it was only to sleep. During the exchanges, he admitted that his wife was making his life hell. He wept as he explained the suffering he endured living alongside his wife. The wife sat in silence, unable to say a word. The man said he could not go on, that she could keep the house and everything in it. We have known of cases of suicide in the past. Violence against men is very real, even if it is not widely known; people do live through it.

Certain male victims of domestic violence do not leave the household for fear of social judgement (27.27%), shame (25.28%), concern for their children’s welfare in the event of separation (20.45%), love and attachment to their partner (18.61%), financial dependence (3.27%), a desire to save the marriage (1.14%), or the hope that their partner’s behaviour will change (1.28%). A small proportion had no opinion on the question of remaining with the aggressor (0.28%). The percentages in this second set of figures are shares of the 704 responses recorded for this multiple-response item, not shares of respondents; they indicate the relative weight of each reason among the reasons given rather than the proportion of men citing it (see the note to Table 7).

## 4 Discussion

Analysis of the socio-demographic results shows that domestic violence was reported across diverse sociodemographic profiles in this sample, spanning different age groups, occupational statuses, educational levels, and religions, suggesting it was not confined to a single social group, although comparative analyses across these groups were not conducted (Gilloz et al., 1999; Brenot, 2008; Hirschelmann, 2020). Even so, the prevalence of 64.55% found here is high compared with international estimates and should be read in light of the convenience sampling used, since it describes the surveyed sample and should not be generalised to all men in Dédougou, or in Burkina Faso, without confirmation from probability-based designs. The broad, behaviourally inclusive definition of violence used here, covering verbal, psychological, economic, sexual, and physical forms experienced at any point in the current relationship, may also have contributed to the high figure observed.

Men are victims of verbal, psychological, economic, cultural, sexual, and physical violence (assault and battery). However, men are disproportionately subjected to verbal violence (Nguema Metogo et al., 2016) compared to other forms. In the present context, verbal violence constitutes a deliberate act on the part of the female partner, intended to affect the man emotionally or to exert a negative impact on the victim (Lindsay and Clément, 1998). Indeed, psychological and verbal violence leave no visible marks upon the body, yet they wound the spirit. The words exchanged during disputes cut through the heart and soul like a blade. Such words are ‘poisoned arrows’, and the mouth the ‘quiver’, in reference to respondents’ proverbs. According to Jaspard (2005), if words can ‘kill’, silence represents another form of violence that society imposes upon male victims. Furthermore, this violence is not confined to the domestic sphere, as it extends into the public domain with the intention of persecuting the victim. The perpetrator exercises a dual form of violence by pursuing this behaviour in the public sphere deliberately, to harass and humiliate the victim, thereby placing the professional identity of the partner in jeopardy (Kopp et al., 2008).

The predominance of verbal and psychological violence found in Dédougou fits with recent psychological research showing that psychological abuse should not be treated as a single, secondary category. Lortkipanidze et al. (2025) find that distinct forms of abuse, verbal-emotional, dominance-isolation, economic, sexual, and physical, have different effects on depression, anxiety, and life satisfaction, and that victims typically experience several forms at once. This pattern of cumulative, multiform victimisation also emerges from the accounts of respondents in this study, where verbal, psychological, and economic violence frequently co-occur and where anxiety and diminished self-esteem stand out among the reported consequences.

Furthermore, these data reveal that men are also victims of physical violence, which represents 9.20% of the surveyed sample. These men are struck, pushed, slapped, scratched, and bitten (Ripkah, 2021). This sheds light on the reality and recognition of the concept of the ‘battered man’. Indeed, as Archer (2000) notes, the prevalence of recourse to physical violence was *d* = 0.5 among women and *d* = 0.15 among men. This study reveals that the violence reported is, for a substantial minority of respondents, recurrent rather than isolated: 34.98% of victims (135 of 386) reported violence occurring frequently or very frequently, and 39.12% (151 of 386) reported violence lasting for more than 1 year (Table 4). More than their identity, it is the masculine roles that are violated and humiliated in the battered man (Torrent, 2001). These ‘emasculated’ men develop suicidal ideation and, in the worst cases, ultimately take their own lives. In addition, 11.54% of the sample had previously experienced sexual intercourse without their consent (Nguema Metogo et al., 2016). These two forms of violence remain taboo and engender mutism among men. The virility and strength that characterise men in patriarchal society are profoundly ill-suited to the experience of being a victim of domestic violence. Within society, sexual violence against men remains a myth and is not formally recognised (Welzer-Lang, 2009). Fear, shame, social judgement, social disqualification, and the aspiration to maintain family unity (Bontoux et al., 2020) mean that men who are victims of sexual and physical violence rarely resort to the competent institutions to report their partner. This ‘humiliating violence’ (Gaulejac, 1996) renders the victim vulnerable and induces profound changes in the construction of their identity. Most male victims struggle to articulate their difficulties and to seek help (Abed et al., 2016), which explains the low rate of recourse to the defence and security forces, three of the 386 men who reported violence, that is 0.78% of victims and 0.50% of the full sample of 598 respondents. Consequently, friends and relatives emerge as the primary resource in the event of domestic violence. Compared to the findings of a survey conducted in four regions of Burkina Faso, men are more likely to turn to friends or relatives, at 34.20%. The man’s own family and the wife’s family are consulted in 2.33 and 1.30% of cases, respectively, when men experience violence (Mejia, 2014). These findings reflect victims’ perception of repressive institutions such as the police, gendarmerie, and judiciary. For these individuals, the operational approach of such modern institutions to conflict management is misaligned with the traditional conflict resolution mechanisms, which are generally more conciliatory, hence the expression: ‘a bad settlement is better than a good lawsuit’. Resorting to such institutions is synonymous with a complete rupture of the marital bond within society. It leaves little scope for reconciliation or conciliation between partners. Moreover, recourse to the police, gendarmerie, judiciary, and social services is perceived as humiliating. The internalisation of masculine norms demands that the man remain dominant and discreet. This pattern is consistent with the concept of hegemonic masculinity (Connell and Messerschmidt, 2005), which describes a culturally idealised standard of manhood built on strength, self-reliance, and emotional restraint; disclosing victimisation directly contradicts this standard and is therefore experienced as a further threat to identity, on top of the violence itself. This helps explain why, in the present study, men who did speak out described being met with disbelief or ridicule rather than support, and why friends and religious leaders, who allow for discreet, face-saving forms of help-seeking, are consulted far more often than police or health services, which require a public acknowledgement of victim status. These gendered patterns of help-seeking match recent findings in the sociology of victimisation. Analysing a representative German sample, Trafford and Le (2025) found that the sources of support people turn to differ markedly by gender, with women relying more on informal networks and male victims confiding more often in health practitioners, and that early recourse to friends and family is associated with shorter durations of abuse. This fits with the central role of friends and relatives (34.20%) observed in Dédougou and suggests that strengthening the response capacity of informal networks could be a particularly effective entry point for intervention. The harsh social condemnation of violent women documented in our qualitative material also has an experimental counterpart, since Zedlacher and Yanagida (2023) show that female perpetrators of ambiguous mistreatment attract more moral anger and stronger attributions of intent than male perpetrators, which supports the interpretation that the figure of the violent woman violates prescriptive gender stereotypes and is consequently seen as deviant, a mechanism that, paradoxically, also silences her male victim, whose disclosure would expose both partners to social disqualification.

According to other scholars, this silence is also attributable to the fact that men, in addition to being the victim, are perceived and treated as the aggressors. The violence they endure is not considered as serious as that experienced by women (Dutton, 2006; Dutton and White, 2013). Violent women are regarded as victims, whilst men who are victims are arrested and presumed guilty (Ripkah, 2021). For some, this violence is justified as a defensive reaction to the man’s initial aggression (Nabila, 2006). In reality, few studies have examined the dominant frameworks that have historically centred female victimisation, despite evidence that complicates this framing (Ripkah, 2021).

Recent epidemiological work supports this more nuanced view. In a study of 5,385 men, Leiding et al. (2022) show that victimisation and perpetration frequently overlap within the same individuals over the life course, and that men in this overlap have the poorest mental and psychosomatic health outcomes. These findings support treating violent dynamics as a continuum rather than through a fixed perpetrator–victim binary (Gueta and Ben-Porat, 2023), a view consistent with Dallaire’s (2002) account of mutual escalation, and point to screening and intervention approaches that address victimisation and perpetration together rather than separately.

The most frequently reported contributing factors are of a socio-economic and moral nature. Another factor reported by respondents is the failure of men to honour their commitments to their partner, particularly fidelity. Respondents most frequently cited economic difficulties as a perceived contributing factor to violence; a descriptive cross-sectional design of this kind cannot establish a causal relationship between income and the occurrence of violence. Economic problems (59.20%) and jealousy or infidelity (52.51%) are the factors most frequently identified by respondents. This sensitivity of violent dynamics to economic standing and to power differences within the couple fits with experimental work in the sociology of intimate relationships showing that tolerance of partner coercion varies with people’s perceived status and position within the relationship (Blake and Brooks, 2018). It supports the view that violence against men in Dédougou is bound up with ongoing renegotiations of economic roles and conjugal power, particularly when the man cannot fulfil the provider role assigned to him by patriarchal norms, rather than with any inherent characteristic of either sex. However, according to Hirschelmann (2020), age and sex are not individual explanatory variables for the occurrence of domestic violence; rather, these variables serve to categorise the groups of individuals most exposed to a high risk of violence.

These findings have practical policy implications for gender-inclusive IPV responses in Burkina Faso and similar resource-constrained settings. Feasible measures would include confidential, non-stigmatising reporting mechanisms within the police, gendarmerie, health, and social welfare services, training for service providers to recognise and respond to male victimisation without prejudice, clearly defined referral pathways linking religious and customary mediators, currently the first recourse of most victims, to formal psychosocial, medical, and legal services, and public awareness campaigns aimed at destigmatising disclosure. Such measures should be framed as an extension of existing IPV services toward gender inclusiveness, not as a shift of resources or attention away from services for women, who remain disproportionately affected by IPV worldwide. The qualitative findings support this reading and help explain why men remain silent and why help-seeking is so low, since disclosure threatens masculine identity and exposes victims to ridicule, disbelief, and the label of ‘coward’ or ‘pushover’, social norms cast the violent woman, not the victimised man, as the deviant figure, so that speaking out shames both partners, and formal institutions are seen as punitive and incompatible with conciliatory, discreet conflict-resolution norms. Interventions therefore need to be culturally appropriate, confidential, and trust-building if they are to reach male victims. These proposals are in line with the recommendations made by Landa-Blanco and Mejía Sánchez (2025) for low- and middle-income countries, namely gender-inclusive service design, professional training to counter disbelief and stigma, and the integration of male victimisation into IPV surveillance systems, and with the call from Rollè et al. (2019) for evidence-based interventions attentive to the full diversity of victim–perpetrator configurations.

These findings should nonetheless be interpreted within clear methodological boundaries. First, because participants were recruited through non-probabilistic convenience sampling rather than random selection, men who were more available, more accessible to enumerators, or more willing to discuss their intimate relationships may be over-represented, introducing a possible selection bias; the prevalence and factor estimates discussed above describe this sample and should not be extrapolated to the wider population of Dédougou or Burkina Faso. Second, given the strength of masculine norms discussed throughout this Discussion, self-reported victimisation is also likely to be affected by social desirability bias and underreporting: men who most strongly internalise the expectation to appear strong and self-sufficient may be the least likely to disclose violence at all, whether to an enumerator or an interviewer, which would tend to understate rather than inflate the true extent of the phenomenon. These and other limitations are set out more fully below.

## 5 Limitations

The findings should be interpreted within seven limitations, concerning sampling, self-report, the instrument, the operationalisation of the object of study, the qualitative analysis, the study design, and the denominators.

### 5.1 Sampling and generalisability

The 598 men were recruited through convenience rather than probability sampling. With no sampling frame, no confidence interval or margin of error can be calculated, and the proportions describe the surveyed sample only. Allocating enumerators across the seven sectors of the municipality improved geographical spread but did not randomise selection; men who were available or willing to discuss their relationships may be over-represented, in a direction that cannot be determined. The results cannot be extended to rural areas of the Boucle du Mouhoun region or to other regions of Burkina Faso.

### 5.2 Self-report, social desirability, and recall

The sensitivity of the topic, self-reports may be affected by social desirability bias. The norms documented by the study (pressure to appear strong, 52.34%; fear of stigmatisation, 64.05%) probably also operated during data collection, tending to depress the observed figures. No independent source (medical, social welfare or police records) was used for triangulation. Because respondents reported on the whole of their current or most recent relationship rather than a fixed recall window, exposure periods vary and recall error is likely; these within-relationship lifetime figures are not comparable with the 12 month prevalence estimates common in the literature.

### 5.3 Absence of a validated instrument

The questionnaire was developed by the author, as no instrument validated for this setting was available; its reliability and validity have not been established, pre-testing having addressed only clarity and cultural appropriateness. The prevalence of 64.55% therefore cannot be compared directly with figures from standardised instruments such as the Conflict Tactics Scales. Moreover, oral translation into Dioula or Bwamu was done without back-translation, so the semantic equivalence of items across languages is not established.

### 5.4 Domestic violence and marital conflict not distinguished

The distinction drawn in the Introduction (Vasseur et al., 2020) between domestic violence, marked by an asymmetry of power, and marital conflict between equals was not operationalised: no item measured power asymmetry, coercive control, intentionality, initiation or reciprocity. Classification as a victim rests on at least one reported violent behaviour, so the 64.55% figure almost certainly aggregates situations ranging from isolated conflicts to sustained coercive control. Frequency (34.98% frequent or very frequent) and duration (22.28% over 3 years) data in Table 4 suggest a recurrent pattern in a substantial minority but do not substitute for a direct measure. The prevalence reflects reported exposure to violent behaviours, not coercive intimate partner violence in the strict sense.

### 5.5 Single-coder qualitative analysis

Coding was carried out by the author alone, with no second coder, no predefined coding frame, no inter-rater reliability statistic and no respondent validation; interpretive bias, mitigated by iterative re-reading and verbatim extracts, cannot be excluded. The 26 purposively selected interviews (four with male victims) illustrate the survey findings rather than establish how widely the views are held. The 11 women interviewed were not the partners of surveyed men, so no relationship is seen from both sides, and women identified as perpetrators are not represented.

### 5.6 Cross-sectional design and causal inference

Data were collected at a single point in time (June–July 2025), with no comparison group and univariate analysis only, without significance testing. No relationship can be interpreted causally: the factors in Table 8 are perceived contributing factors reported by all 598 respondents, including the 212 non-victims, and the consequences in Table 7 are attributed to the violence by respondents themselves. Confounding and reverse causation cannot be excluded; the mechanisms proposed in the Discussion are hypotheses that would require longitudinal, comparative or dyadic designs.

**Table 8:** Reasons for domestic violence.

Value	Frequency	Percentage (%)
Economic problems	354	59.20
Infidelity or jealousy	314	52.51
Conflicts over power or respect within the couple	216	36.12
Cultural misunderstandings	129	21.37
Influence of the wife’s family	127	21.24
Alcohol or drug consumption	103	17.22
Witchcraft	53	8.86
Curses	50	8.36
Lack of communication	6	1.00
Irascible character	2	0.34
Sexual dissatisfaction	2	0.34
Negative social influences	1	0.17
Forced marriage	1	0.17
Violence against children from a previous marriage	1	0.17

Source: Survey, June–July 2025. These questions were put to all 598 respondents, including the 212 who did not report victimisation; the percentages therefore reflect perceived contributing factors to violence in general rather than the factors respondents associated with violence they had themselves experienced, and multiple responses were possible.

### 5.7 Denominators and scope

Denominators vary across tables. Tables 3, 6–8 (first block) use the 598 respondents, and multiple responses sum to more than 100%; effects are thus not proportions of the 386 victims. Table 7 (second block) uses the 704 responses recorded, and only Table 5 uses the 386 victims. This calls for care in comparisons with the literature, which generally uses respondent-level denominators. Finally, the study measured male victimisation only, with no parallel measurement of violence against women or perpetrated by the respondents, and supports no comparative claim about men’s and women’s experience of intimate partner violence in Dédougou.

## 6 Conclusion

This study aimed to determine the forms, perceived contributing factors, and psychological and social consequences of domestic violence against men. It highlights an often overlooked reality, that of domestic violence against men. Far from being a marginal phenomenon, this form of violence was reported by 64.55% of the men surveyed in this convenience sample, across diverse social, occupational, and religious profiles; because the sample was not drawn probabilistically, this proportion should be read as descriptive of the respondents rather than as an estimate for men in Dédougou or Burkina Faso more broadly. Such domestic violence takes diverse forms, including verbal, psychological, physical, sexual, economic, and cultural violence. Shame, fear, social disqualification, and the weight of masculine norms compel these victims to withdraw into silence. The low recourse to qualified protection and enforcement services is linked to the inadequacy of modern institutions in relation to the traditional mechanisms of conflict resolution between partners, which tend to be more discreet. Furthermore, the lack of social recognition of physical and sexual violence against men in intimate partnerships contributes to rendering the phenomenon invisible and to perpetuating the myth of male invulnerability. The factors most frequently reported by respondents were economic, with jealousy and infidelity as significant additional factors. The consequences are manifold, potentially including depression, stress, anxiety, and, in the most serious cases, suicide. These findings challenge the dominant discourse that designates the man as the sole aggressor within the couple. They accordingly call for an inclusive policy, support for victims, formal acknowledgement of this masculine suffering, and encouragement of recourse to protection mechanisms. By documenting the prevalence, forms, causes, and consequences of IPV against men in a West African context, and by shedding light on the normative mechanisms that sustain the culture of silence, this study also speaks to inclusive IPV research, policy, and intervention strategies in resource-constrained settings, including the surveillance and measurement of under-recognised forms of IPV, confidential reporting pathways, provider training, psychosocial support, and culturally appropriate, gender-inclusive services. Recognising male victimisation in intimate partnerships does not diminish the gravity of violence against women; it completes the picture of IPV and strengthens the collective capacity to prevent and respond to violence in all its forms.

## Data Availability

The raw data supporting the conclusions of this article will be made available by the author, without undue reservation.

## Footnotes

1 This expression means that the words that come from her mouth (compared to a quiver) are like poisoned arrows.
